# Prevalence of musculoskeletal injury and pain of UK-based podiatrists and the impact of enforced altered working practices

**DOI:** 10.1186/s13047-021-00491-7

**Published:** 2021-09-01

**Authors:** Robert Adams, Helen Branthwaite, Nachiappan Chockalingam

**Affiliations:** Centre for Biomechanics and Rehabilitation Technologies, Stoke on Trent, Staffordshire University, Leek Road, ST4 2DF Stoke-on-Trent, UK

## Abstract

**Background:**

Occupational musculoskeletal injuries are prevalent in healthcare workers and are reported to be profession-specific. There is, however, a paucity of information around the injuries sustained from working as a podiatrist. This paper looks at the incidence of injury from working as a podiatrist, the aggravating factors to sustain these injuries and whether the changes in workload due to the COVID-19 pandemic altered the incidence.

**Methods:**

A modified work based musculoskeletal injury questionnaire was distributed in the UK via podiatry led social media platforms. Open and Closed questions explored the demographics of the sample, perceived injury 12 months prior to the COVID-19 pandemic and then 6 months into the lockdown. Pre and post COVID-19 data were analysed for differences and thematic analysis was included to categorise reported experiences.

**Results:**

148 podiatrists representing 3 % of HCPC registered practitioners responded to the questionnaire. Employment status altered as a result of the COVID-19 pandemic with a 13 % reduction in those working full time. Environments also changed with domiciliary and telehealth significantly increasing (p > 0.00) and non-clinical roles being extended (*p* > 0.002). Pain frequency and intensity significantly (*p* > 0.04) increased as a result of the pandemic with shoulder pain being most frequent before lockdown altering to the neck during the lockdown. Two main themes were identified that were attributed to the causes of pain including physical demands and working in awkward spaces.

**Conclusions:**

Work-related musculoskeletal pain in podiatrists is common with the shoulder and neck being the most frequently affected. Changes in work practices due to the restrictions enforced from the COVID-19 pandemic increased the frequency and intensity of pain mostly associated with increased domiciliary and telehealth working environments.

**Supplementary Information:**

The online version contains supplementary material available at 10.1186/s13047-021-00491-7.

## Background

Healthcare professionals have one of the highest work-related musculoskeletal (MSK) health problems in the UK workforce, with 66 % reporting injury [1, 2]. This leads to increased use of healthcare services, loss of workdays and reduced productivity [3]. Anatomical regions injured appear to be specific to the profession with reports of hand pain in Physiotherapist and shoulder problems in Sonographers [4, 5]. When exploring the occupational health risks of Podiatry there is an indication that 76 % of Podiatrists suffer from MSK injury [6], with the most frequently affected area being the lower back [7, 8] and the hand being observed due to instrument use [9]. There is however a lack of understanding of how these problems impact practice and what changes podiatrists make to address work-based MSK pain. Exploring this area further will help to inform policy and current guidelines for practice.

MSK pain has been linked to the ergonomic challenges of working as a Podiatrist and can arise from a lack of ideal ergonomic positioning and working aids [10]. Accessing the foot at the appropriate height and angle can stretch clinicians into positions that increase the load on joints and incur repetitive movements. In a clinical setting, the working position can be improved with the use of appropriate hardware such as the patient couch, clinician chair and operating trolley. However, these are not an option on a domiciliary basis, which has been shown to challenge posture with excessive flexion of the neck and a slumped position [8]. During domiciliary treatment podiatrists are often in cramped, awkward positions on the floor or at the end of a bed. There can be a little variation of posture, which can result in specific MSK demands on the body [6].

Podiatry clinics had to alter service delivery overnight during the COVID-19 pandemic. Frequencies of care provision changed as continuation of service was critical whilst limiting patient contact. Telehealth became commonplace [11, 12] and strict guidance on suitable patient contact was made [13]. These changes impacted working practices and altered the way that Podiatry was being used as a service and delivered [14], as well as many podiatrists being redeployed into non podiatry roles. The long-term impact of these changes has yet to be established as the pandemic continues. This change in practice gave an opportunity to investigate MSK work-related injuries sustained from working as a podiatrist prior to and during a 6 month period of the COVID-19 pandemic, March 2020.

## Methods

Purposive sampling of UK, HCPC registered podiatrists was employed to engage in an online questionnaire, Qualtrics® (Prov, UT, USA) previously utilised to evaluate MSK injury [6]. The questionnaire was however modified to reflect changes in practice associated with the COVID-19 pandemic, specifically to explore how working practices had changed and links between risk factors. This included questions on the kind and quantity of work clinicians had been doing both before and during the pandemic, and wider demographics (Additional information 1). Questions included open and closed design to allow participants expression of experiences on MSK injury. The changes made were piloted before final distribution. Ethical approval was sought and obtained from the University Ethics Committee and adhered to throughout the study with all data collated from the questionnaire anonymised at source.

### Participants

Participants were targeted using online professional podiatry forums, both MSK-specific and for the profession as a whole. Following administrator consent, the study was advertised on social media groups including MSK:UK and Podiatry-UK, and additional external networking through local podiatry branches. G-Power 3.1 software, a Wilcoxon signed rank test [15, 16] predicted a minimum sample size of 57 participants required, in order to provide results with a power of 0.95 with 0.05 error. For responses to be included in the research, participants had to meet the following criteria:

- to be a practising UK-based podiatrist.
- have at least some percentage of their workload based around service provision to patients.
- to have continued to have worked in a healthcare setting throughout the pandemic, whether this be within podiatry or from redeployment.

Participants were excluded if they failed to meet the above criteria or failed to complete the questionnaire to the end and omitted some sections or individual questions.

### Data Collection

The questionnaire was live with regular re-advertising for 12 weeks which allowed the results to be gathered with an exact 6-month retrospective reflection of practice post-COVID-19 (March 2020 – Sept 2020) compared to a 12-month period of normal working practices directly prior to this (March 2019 – March 2020).

### Data Processing

Representation of the participating podiatrists was collated to provide demographic details. Quantitative findings related to MSK pain whilst practising podiatry were anlalysed using IBM SPSS statistical software. Data were catagorised into pre and post COVID-19, to capture the impact of enforced environmental changes on clinical practice. The frequency of pain occurrence were analysed using McNemar’s test whilst parametric data were subjected to a paired samples t-test. Significance was based on a confidence of 95 % *p* > 0.05.

Qualitative data on perceived risk factors were collated into themes which allowed inclusion and comparison between pre- and post-COVID-19 conditions.

## Results

There were 148 respondents of which 85 % were female and 15 % male, dominated by white ethnicity 93 %, Asian 2.7 % and mixed race 2 %. Age was evenly distributed with 20 participants over 55 years, 83 aged between 40 and 54, 31 participants aged between 30 and 39 and 14 under aged 30. Over half of respondents had been practising for over fifteen years, with this accounting for 57.4 % of the group with 12 % were from newly qualified clinicians who had been practising for less than five years.

60 % of participants reported no underlying health issues, whilst the remainder reported to have cardiac and breathing difficulties (*n* = 5.6 %) inflammatory arthritis (*n* = 3.5 %), mental health (*n* = 2.8 %) Osteoarthritis (*n* = 5.6 %) and 15.4 % having co-morbidities, remaining 7.1 % preferred not to say.

Before the COVID-19 pandemic, 51.4 % of participants worked full time, 33.8 % 4 days a week, 12.8 % 3 days a week and 2 % 2 days a week. Once lockdown occurred in March 2020 this changed to 38.5 % Full time, 27.7 % 4 days per week, 14.9 % 3 days per week, 6.8 % 2 days per week, 4.1 % 1 day per week and 8.1 % less than one day per week. Working environments altered also with work in domiciliary settings increasing significantly during the pandemic as well as non-clinical roles and telehealth. Community NHS clinic work significantly reduced and hospital consultations remained the same. (Table 1).

**Table 1:** Average percentage workload of participants in each environment pre COVID-19 and during the 6 months data collection. Significance highlighted from paired sample t-test *p > 0.05

Working Environment	Pre COVID	During COVID	Significance
Hospital NHS – acute care	8.85	8.38	0.718
Community Health centre NHS	35.52	10.51	0.000*
Private Practice	30.81	25.88	0.004*
Domiciliary	17.54	35.04	0.000*
Telehealth	0.1	6.11	0.000*
Non - Clinical	5.78	10.14	0.002*
Other non-podiatry work	1.4	3.94	0.319

Reported frequency of pain was slightly more during the pandemic than prior with 66 % of participants reporting pain during the pandemic period and 64 % prior to a change in working practices. Location of pain was most frequently reported in the shoulders 39.2 % before COVID-19 then during this altered to the neck 35 %. There were no significant differences in the location and frequency of pain during the two time periods (Table 2). However, the numerical score rating the severity of pain increased significantly (pre 3.39 +/- 2.73 post 3.89 +/- 2.95 *p* > 0.04) and the frequency of pain increased with daily occurrence of pain rising from 30 % of respondents to 36.5 % and those reporting no pain decreasing during the pandemic from 31 % pre to 25 % during.

**Table 2:** Reported frequencies of pain in each anatomical region represented as a percentage pre COVID-19 and during the 6 months data collection. Significance highlighted from McNemar’s test *p > 0.05

Anatomical Location of Pain	Pre COVID %	Post COVID %	Significance *p* > 0.05
Lower Back	27.7	32.4	0.296
Mid back	3.1	12.2	0.064
Upper Back	13.5	14.2	1
Shoulders	39.2	31.8	0.08
Neck	33.1	35.8	0.644
Elbow	5.4	6.1	1
Hand	19.6	15.5	0.345
Thumb	9.5	10.1	1
Hip	4.7	8.8	0.109
Knee	7.4	12.8	0.096
Ankle	6.8	9.5	0.344

Exploring and anlaysing the open-ended questions allowed for rich data to be extracted regarding painful injury and the experiences participants had from working with modified clinical practices during COVID-19. This data resulted in two main themes emerged that were categorised as:

1. Physical Demands.
2. Awkward working spaces with poor ergonomics.

### Physical Demands

Domiciliary work was highlighted as the biggest cause of pain. 58 % of the participants who made home visits reported pain before the pandemic. This raised to 60 % during COVID-19. Transporting equipment in and out of patient’s homes or care homes and increasing driving were identified as activities that caused the problem.

*Participant 133* stated “I developed knee pain from driving for dom visits and the positions I sat in during the visits” and Participant 33 highlighted “Mid back pain due to seeing more domiciliary and ward patients with reduced help from ward staff to reduce exposure from COVID-19”.

This practice increased after the COVID-19 lockdown as did computer based work, which was also highlighted as an issue for pain as participants commented on how they had suddenly changed from being in a clinic, walking around to sit just at a computer.

*Participant 72* stated “I’m not a sitting at a desk holding the phone or at the keyboard type person. I struggle to remember to move. Having patients in *[the]* clinic kept me more mobile”.

Participants reported both extremes of movement, heavy lifting and sitting still for long periods, as the main causes of work-related pain. The changes in practice due to COVID-19 appeared to exacerbate both activities and participants described the pain post COVID-19 to be more intense, stiffer and constant.

*Participant 21* “More constant pain in left neck shoulder and arm hand now, prior to covid did not get constant pain in hand and arm occasionally had flare ups with neck and shoulder only”.

### Awkward working spaces with poor ergonomics

Pain related to working positions that are required to complete the job of a podiatrist were also singled out as a factor contributing to MSK problems. Working outside of the clinical setting was indicated as a key concern for many with participants reporting to work in very cramped and awkward conditions.

*Participant 10* “Bending or twisting your back in an awkward way, Lifting or transferring dependent patients all add to pain”.

Domiciliary bags, computer chairs and desks were all singled out as persistent challenges for participants.

*Participant 127* “I have to wear a support on my knee and elbow to carry my Dom bag”.

Sudden changes in practice due to COVID-19 were also singled out as consultations moved to telehealth.

*Participant 117* stated “Longer periods and instances of telephone triage, sitting at a desk or at home on a small laptop” had increased lower back pain.

When asked about a DSE screen assessment most participants did not know what the assessment was and had not been able to access any services to evaluate the working environment. In total only 15 % had engaged in this assessment to improve working practice.

## Discussion

As shown in this study, working as a podiatrist is physically demanding, with MSK pain associated with working practices being commonplace. Multiple anatomical regions are affected with the neck, shoulders and lower back being equally shared as the most problematic areas. This work supports previous studies on MSK injury in podiatry[6–8] and also aligns with problems observed in other healthcare professions [4, 5]. This study additionally highlighted that changes in working practices enforced due to the COVID-19 pandemic altered the reported incidence of MSK pain as podiatrists worked less in community health centres and attended more domiciliary visits. Although the restrictions and guidance from the UK government advised against non-essential healthcare in the first phase of the pandemic, March 2020 [17], red category patients who had active ulcers or were at high risk of developing a complex foot problem continued to be cared for [13] moving consultations into the patients home.

Podiatry treatment is provided for patients in multiple settings with NHS community clinic provision and private practice being reported most frequently within this group before the COVID-19 pandemic. These environments include ergonomic hardware such as the patient couch, clinician chair and operating trolley which allow for adjustments in height and position. Having a flexible working environment is strongly recommended to dentists who most commonly retire due to work associated MSK problems [18]. Restricting this environment during the 6-month COVID-19 pandemic with work altering to domiciliary and telehealth could have been attributed to the increase in pain as demands on the body alter. The posture of podiatrists is poor, especially when providing domiciliary care as the treated foot is often placed on the podiatrist’s knee causing excessive flexion of the neck [8]. This posture is also associated with increased trapezius muscle activity and is associated with tasks such as computer work [4], with forward flexion of the neck being linked to back problems [19]. Additionally, the change from community health clinics to domiciliary work, either in the patients own home, care home or with assisted living, reduces the number of contacts made per day increasing the waiting times for patients and reducing the economic efficiency of the service.

When looking at injury and pain reported as a consequence of working as a podiatrist the shoulder was reported most frequently prior to the pandemic which then changed to the neck during to 6 month COVID-19 period. Although these changes were not significantly different, they are both associated with forward flexion of the neck seen in podiatry practice. Interestingly, the most common ergonomic challenge reported in dentists was prolonged maintenance of position and forward flexion of the neck or trunk, with upper arm elevation being an additional high MSK risk for shoulder pain. It was discovered that an increment of just 1 % upper arm elevation over the working day could be linked to tendinitis and shoulder pain [2]. Although working on different regions of the body, dentists adopt a similar position to podiatrists when completing their work. Therefore considerable attention should be paid to arm and neck position when treating podiatry patients to avoid work related neck and shoulder pain. A specific work based ergonomic assessment of podiatrists posture during intervention is recommended particularly when completing domiciliary care. Although it is not the purpose of this work to review the HSE guidance [20] on working practices for podiatry in a domiciliary setting, reporting the incidence of MSK problems in practice highlights that despite following rules and regulations still results in worked based injury. Advancing training in the ergonomics of practising safely would assist podiatrists in completing their role, as seen in other healthcare professions [21].

From the thematic analysis completed, participants experiences of pain and the physical demands of being a podiatrist shone through, with domiciliary work being most attributed to pain. This was associated with transporting equipment, heavy lifting and driving. Where on the other end of the spectrum changes due to COVID-19, sitting at a desk was thought to increase pain. It is well established that long periods of sitting can be detrimental to MSK health and short periods of seated computer work are recommended [22]. Additionally, altered inactivity was highlighted as an issue, as walking around a clinic and attending to patients were replaced with days sat at a desk. Although it is deemed mandatory to have a DSE screen assessment, it is clear from this work that this is not often adhered to, leaving podiatrists at risk. This could have been as there was a rapid change in practice leading to a lapse in protocol. Prevention of work related injuries in nursing are effective with an exercise program where conditioning workers to the tasks involved in the role reduced the incidence of pain [23]. This type of prevention should be included in conditioning podiatrists with neck and shoulder strengthening and exercise programmes being implemented which could reduce the injuries sustained from practising.

Conditioning podiatrists to work with repetitive movements and heavy lifting would not resolve the cramped and awkward working spaces and positions reported to be associated with work-based pain in podiatry. Focusing on appropriate equipment to deliver domiciliary care would be most appropriate to assist podiatrists in attending to patients in their own home. To date, there is no research to support the use of specialist foot stools and operating chairs to use in podiatry domiciliary work that would reduce work related pain. Guidance on how podiatry can be safely implemented in a patients home is required to give support to those working in this environment. A thorough review of the equipment required to effectively treat a domiciliary case without undue injury is also essential so that the incidence of work place MSK injury is lowered, improving the wellbeing and lifespan of those working in podiatry.

Although the gender bias within the participant characteristics could be seen as a limitation, this is not too dissimilar to the gender distribution of the UK podiatry profession. The female to male ratio at the last Health and Care Professions Council registration being 76.2 %:and 23.8 % [24]. Therefore, the results are indicative of the demographics of the profession. The difference in the two data collection time periods (12 and 6 months respectively of pre- and post-COVID-19) needs to be acknowledged. Whilst it would have been ideal to keep same time periods, extending the post-COVID-19 time period was not feasible, due to the rapidly changing advice from Government and other healthcare agencies. Also, on reflection, the data could have been enhanced with a greater specification of injury detail(s) within the questionnaire. This would have provided further details on exact location and diagnosis of the injury.

## Conclusions

MSK pain related to work is commonplace in podiatry practice. The shoulder and neck are most affected associated with a forward flexed neck posture adopted to treat patients’ feet. COVID-19 pandemic enforced a change in working environments seeing care move away from community clinics and increased domiciliary care but did not significantly alter observed pain. Domiciliary care and heavy lifting are most associated to pain and specialist equipment designed to improve working in this environment are recommended. A review of policy and procedures around safe working to support those who suffer from work based MSK pain is required to limit shortened careers and improve wellbeing.

## Supplementary Information

**Additional file 1.**

## Data Availability

Please contact author for data requests.

## Abbreviations

MSK: Musculoskeletal
UK: United Kingdom
HCPC: Health and care professions council
NHS: National Health Service
