# Supplementary material for: Prevalence of musculoskeletal injury and pain of UK-based podiatrists and the impact of enforced altered working practices
Source: J Foot Ankle Res. 2021 Sep 1;14:53. doi: 10.1186/s13047-021-00491-7 (PMC8409074; doi:10.1186/s13047-021-00491-7)
Supplement: Supplementary file 1 — Additional file 1. [file 13047_2021_491_MOESM1_ESM.pdf]

Additional file 1 – Qualtrics questionnaire:

Participant background information:

What is your gender?

- Male
- Female
- Prefer not to say

How would you identify your ethnicity?

- Asian or Asian British. Includes any Asian background, for example, Chinese, Indian, Pakistani
- Black African/Black British/Black Caribbean. Includes any Black background
- Mixed or multiple ethnic groups. Includes any mixed background
- White. Includes any White background.
- Other. Includes any other ethnic group, for example arabic.
- Prefer not to say

What is your age?

- Under 25
- 26-30
- 31-35
- 36-40
- 41-45
- 46-50
- 51-55
- 56-60
- 61-65
- over 65

For how many years have you been practicing as a podiatrist?

- 0-5
- 6-10
- 11-15
- Over 15

Do you suffer from any of the following: (please select all that apply)

- Inflammatory arthritis (for example: rheumatoid; fibromyalgia)

- ☐ Osteoarthritis
- ☐ Diabetes
- ☐ Endocrine disorders (for example hypothyroidism)
- ☐ Cardiac conditions
- ☐ Breathing conditions
- ☐ Long term mental health issues
- ☐ Other chronic health issues
- ☐ Prefer not to say
- ☐ No underlying health issues

If you have answered 'other' please detail further below. If not please answer N/A.

Working practices and injury PRIOR to the outbreak of Covid-19

We are now interested in the following:
- your typical working environment **PRIOR** to the outbreak of Covid-19 and
- musculoskeletal pain or injuries experienced while performing your work role **PRIOR** to the outbreak of Covid-19.

For the purposes of this questionnaire, **prior** to Covid-19 is considered to be before the UK Government announced a national lockdown in March 2020.

Questions on how things have altered since the start of the pandemic will be asked in a following section.

We are not interested in pain or injuries that occur as a result of sport or accident external to your work role. For the purpose of this survey, musculoskeletal stiffness or pain due to your work role is viewed as an injury if it results in pain or causes you to change the way you do things at work or home. Please do not include pain that is a result of feverish illness or menstruation.

What has been your typical weekly workload **prior** to the outbreak of Covid-19?

- Full time
- 4 days per week
- 3 days per week
- 2 days per week
- 1 day per week
- Less than one day per week

What has been your typical working environment **prior** to the outbreak of Covid-19? (must add up to 100%)

- 0
Public sector - hospital (treating patients as either outpatients in hospital or those who are admitted as inpatients)
- 0
Public sector - community health clinics (treating patients who come to you as outpatients funded by the NHS)
- 0
Private practice - patients who attend your clinic and pay for their treatment either with or without health insurance
- 0
Domiciliary visits (either public or private sector)
- 0
Distance consultation (for example telephone appointments)
- 0
Non-clinical (includes management, administration, supervision, mentorship and research)
- 0
Other
- 0
Do not currently practice as a podiatrist
- Total

If you have answered 'other', please detail your remaining working environment **prior** to Covid-19:

In the 12 months **preceding** Covid-19, had you experienced any musculoskeletal pain or injury as a result of your work?

- Yes
- No

Please record or estimate the number of instances of pain or injury to each body region in the preceding 12 months to the pandemic that resulted in symptoms lasting for 7 days or longer

	Click to write Column 2<br><br>Answer 1
Lower back	
Mid back	
Upper back	
Shoulders	
Neck	
Elbows/forearm	
Hands/wrist	
Thumbs	
Hips/thighs	
Knees	
Ankles/feet	
Other (please state location and incidence)	

Had the pain or injury you experienced resulted in you having to change your working practice? If yes, please detail how and which body part has caused you to alter your practice. If not, please answer 'no'.

On a scale of 1 – 10, with 1 being no pain and 10 being the worst pain imaginable, how bad is the usual average for the most symptomatic of these reported problems?

- 1
- 2
- 3
- 4
- 5
- 6
- 7
- 8
- 9
- 10

How often did you have the pain?

- Daily
- 2-3 times a week
- Once a week
- Less than once a week

How many days off work had you been forced to take in the 12 months **preceding** Covid-19 as a result of musculoskeletal pain? Please do not include days off related to stress, family matters or other illness

- 0
- 1
- 2
- 3
- 4
- 5
- 6 - 10
- 11 - 15
- more than 15

Is there anything you wish to expand upon or detail further in relation to your pain / injury? If no please answer 'no'

Working practices and injury DURING the Covid-19 pandemic.

We are now interested in the following:
- your typical working environment **DURING** the outbreak of Covid-19
and
- musculoskeletal pain or injuries experienced while performing your work role **DURING** the outbreak of Covid-19.

For the purposes of this questionnaire, **during** the pandemic is considered to be after the UK Government announced a national lockdown in March 2020.

We are not interested in pain or injuries that occur as a result of sport or accident external to your work role. For the purpose of this survey, musculoskeletal stiffness or pain due to your work role is viewed as an injury if it results in pain or causes you to change the way you do things at work or home. Please do not include pain that is a result of feverish illness or menstruation.

What has been your typical weekly workload **during** the pandemic?

- ○ Full time
- ○ 4 days per week
- ○ 3 days per week
- ○ 2 days per week
- ○ 1 day per week
- ○ Less than one day per week

What has been your typical working environment **during** the pandemic? (must add up to 100%)

- [0] Public sector - hospital (treating patients as either outpatients in hospital or those who are admitted as inpatients)
- [0] Public sector - community health clinics (treating patients who come to you as outpatients funded by the NHS)
- [0]

Private practice - patients who attend your clinic and pay for their treatment either with or without health insurance)

- 0
Domiciliary visits - (either public or private sector)
- 0
Distance consultation (for example telephone appointments)
- 0
Non-clinical (includes management, administration, supervision, mentorship and research)
- 0
Other
- 0
Do not currently practice as a podiatrist
- 0
I have been redeployed
- Total

If you have answered 'other', please detail your remaining working environment during the pandemic. If you have been redeployed please detail how/where:

Have you had any musculoskeletal pain or injuries since the start of Covid-19 as a result of your work?

- Yes
- No

Please record or estimate the number of instances of pain or injury to each body region since the UK Government announced a national 'lock-down' in March 2020, that resulted in symptoms lasting for 7 days or longer

	Click to write Column 1<br><br>Answer 1
Lower back	
Mid back	

	Click to write Column 1<br><br>Answer 1
Upper back	
Shoulders	
Neck	
Elbows/Forearm	
Hands/wrist	
Thumbs	
Hips/thighs	
Knees	
Ankles/feet	
Other (please state location and incidence)	

Since the outbreak of Covid-19 how many days off work have you taken as a result of musculoskeletal pain. Please do not include days off related to stress, self-isolation, family matters or other illness.

- 0
- 1
- 2
- 3
- 4
- 5
- 6 - 10
- 11 - 15

- more than 15

Has the pain or injury you have experienced resulted in you having to change your working practice? If yes please detail how and which body part has caused you to alter your practice.

On a scale of 1 – 10, with 1 being no pain and 10 being the worst pain imaginable, how bad has the usual average been for the most problematic of these reported problems?

- 1
- 2
- 3
- 4
- 5
- 6
- 7
- 8
- 9
- 10

How often have you have the pain?

- Daily
- 2-3 times a week
- Weekly
- Less than once a week

Is there anything you wish to expand upon or detail further in relation to your pain / injury? If no please answer 'no'

If you have had musculoskeletal pain or injury since the start of Covid-19, how long has it been since you had a whole month without any pain to this region (please select one option only) If you have more than one MSK complaint, please select for the most problematic region.

- Less than 3 months
- 3 months or more, but less than 7 months
- 7 months or more, but less than 3 years
- More than 3 years

Finally we would like to ask how additional risk factors have changed since the outbreak of Covid-19.

Have your general physical activity levels changed since the outbreak of Covid-19?

- Greatly increased
- Slightly increased
- No change
- Slightly decreased
- Greatly decreased

Have you performed a DSE (display screen equipment) assessment, or has one been performed for you, since working practices have changed with Covid-19?

- yes
- no

If yes, have any risk factors been identified: Please specify. If no assessment has been performed, please enter N/A.

Have any changes been implemented and have these helped? If not please answer 'no'

Which are the main job factors you feel may contribute to musculoskeletal pain and injury for podiatrists? (please select as many as relevant)

- Performing the same task over and over
- Working in the same position for long periods
- Treating a large number of patients in a single day
- Bending or twisting your back in an awkward way
- Lifting or transferring dependent patients
- Continuing to work when injured or hurt
- Reaching or working away from your body
- Working in awkward or cramped conditions
- Not enough rest breaks during the day
- Unanticipated sudden movement or fall by the patient

- ☐ Assisting patient during gait activities
- ☐ Carrying, lifting or moving heavy materials or equipment
- ☐ Working with confused or agitated patients
- ☐ Inadequate training in injury prevention
- ☐ Computer/desk work and administration
- ☐ Remote working

Has your work exposed you to an increased exposure to any of these since the start of the outbreak of Covid-19? (please detail which)

[ ]

Would you describe your pain differently than before Covid-19? For example - "previously it ached, now it stings" If no change other than severity please write 'no'

[ ]

How do you normally manage your symptoms? (Please select all that apply)

- ☐ Analgesia
- ☐ Yoga/Pilates
- ☐ Other exercise
- ☐ Intervention from another health care professional or practice or previously prescribed exercises, eg stretches.
- ☐ Massage
- ☐ Other

Have you had to increase these interventions since the start of Covid19?

- ○ Yes
- ○ No
